# Carbon source utilization patterns in dental plaque and microbial responses to sucrose, lactose, and phenylalanine consumption in severe early childhood caries

**DOI:** 10.1080/20002297.2020.1782696

**Published:** 2020-06-23

**Authors:** Weihua Shi, Jing Tian, He Xu, Guiyan Wang, Qiong Zhou, Man Qin

**Affiliations:** Department of Pediatric Dentistry, Peking University School and Hospital of Stomatology, Beijing, China

**Keywords:** Severe early childhood caries, carbon source utilization, metabolic microbial profile, oral microbiome, sucrose, lactose, phenylalanine

## Abstract

**Background:**

Severe early childhood caries (S-ECC) is mainly caused by the interaction of microbiota and environmental factors. However, the metabolic profiles of S-ECC microbial communities and the community-level microbial responses to carbohydrates and amino acids are poorly understood.

**Methods:**

We collected supragingival plaques from 15 caries-free (CF) and 14 S-ECC children. Cultivation on Biolog AN microplates together with next-generation sequencing was used to analyze sole carbon source utilization patterns and microbial responses to sucrose, lactose and phenylalanine.

**Results:**

S-ECC plaques had greater overall metabolic activity than those of CF ones. Comparing with CF, S-ECC plaques utilized more sucrose and lactose but less phenylalanine and then had greater response to carbohydrates. A remarkable increase of non-mutans Streptococci was observed in sucrose and lactose consumption. Lactose led to less differently distributed taxa than sucrose in both CF and S-ECC groups. Sucrose made the originally different S-ECC and CF communities eventually became similar to each other, but they remained dissimilar in lactose.

**Conclusion:**

S-ECC plaques had more active interaction with cariogenic carbohydrates like sucrose and lactose than healthy plaques. We supported lactose has less cariogenicity compared with sucrose from microbial community structural aspect. Phenylalanine may have a potentially inhibitory effect on caries development.

## Introduction

Despite comprehensive approaches being used for prevention and treatment, severe early childhood caries (S-ECC) remains a persistent problem that significantly affects the life quality of the child and their family in both developed and developing countries [1].

S-ECC, like other types of dental caries, is a multifactorial disease that is mainly caused by the interaction of bacteria and environmental factors on tooth enamel. *Streptococcus mutans*, the most intensively studied organism, is strongly associated with S-ECC [2–4]. Recent studies using advanced detection techniques identified that other bacteria, such as *Prevotella* spp., *Lactobacillus* spp., *Dialister* spp., and *Filifactor* spp. [5], genera such as *Streptococcus, Porphyromonas*, and *Actinomyces* [6] and *Scardovia wiggsiae* [7] are also linked with S-ECC. These microbial data suggested that there is not a single pathogen, but rather a pathogenic population that correlates with S-ECC. Therefore, we should adopt an ecological perspective to further investigate its microbiological etiology [8,9]. Previous studies of the interactions of environmental factors with oral bacteria often focused on one or several species. For example, it was suggested that *S. mutans* had evolved elegant strategies to cope with the different carbohydrates present within the oral cavity [10]. Carbohydrates, such as sucrose or lactose, could in turn affect the diversity of genotypes and biofilm formation of *S. mutans* [11,12]. However, our knowledge of community-level metabolic profiles of S-ECC microbial consortia remains limited.

The presence of excess carbohydrates is often responsible for altering the local environment, making it more favorable for species associated with the initiation and progression of dental caries [13]. Among different dietary carbohydrates, sucrose is considered to be the most cariogenic because it can be fermented, induce a low pH environment, and makes the resident plaque microflora more cariogenic. Sucrose can also serve as a substrate for the synthesis of extracellular polysaccharides that promote changes of biofilm matrices in the caries process. Additionally, low concentrations of Ca, Pi, and F were detected in dental biofilms formed in the present of sucrose, which are critical ions in remineralization of enamel and dentin [14,15]. Abundant in milk and formulas, lactose is another important dietary carbohydrate, especially for infants and young children. Decayed lesion formation in hamsters and desalivated rats indicated the cariogenicity of lactose [16,17]. Lactose is fermented to yield organic acids by oral microorganisms, including bacteria such as *S. mutans* and *S. sobrinus*, leaving a low final culture pH, which could begin demineralizing dental enamel and ultimately cause a decaying lesion [18]. The transition in plaque composition between healthy and S-ECC is driven by the bacterial response to environmental changes. Therefore, to obtain a better understanding of the etiology of S-ECC, it is necessary to explore the microbial community responses to sucrose and lactose.

Amino acids, another important carbon source for dental plaque, can serve as substrates for alkali production, which contributes to acid neutralization in the process of dental caries [19], inhibits bacterial coaggregation, and disrupts multi-species cariogenic biofilm assembly [20,21]. Phenylalanine, an essential nutrient for humans, is widely used in food and for the production of the artificial sweetener aspartame [22]. Our preliminary study showed that the microbial community of healthy children had greater metabolic activity in response to phenylalanine than did the caries group. Other studies reported that the concentration of phenylalanine in saliva has a negative association with the decayed, missing, and filled teeth (DMFT) index [23] and dental caries in mice was significantly decreased in the group receiving phenylalanine [24], which also suggested that phenylalanine had an inhibitory effect on caries development. Phenylalanine is essential for the growth of some oral anaerobes [25]; however, whether and how oral microbiota is associated with the effect of phenylalanine on dental caries remains unclear.

Sole carbon source utilization technology, such as Biolog microplates, provides analysis tools to explore a community-level metabolic profile. Biolog microplates are commercially available 96-well plates that include carbohydrates, amino acids, organic acids, nucleotides, and nucleoside. Biolog technology has proven useful to investigate the physiological profiles in patients with periodontitis and children with ECC [26,27]. Each well can also be used as proprietary growth media and allows testing for the responses of the whole ecosystem microbiota to these separate carbon sources simultaneously. In addition, next-generation sequencing techniques make it possible to study the diversity and structure of the oral microbial community and have facilitated investigations into the associations of oral bacteria taxa with caries in children [28–31]. Therefore, in the present study, community-level profiles of microbial consortia were studied using the statistical analysis of Biolog sole carbon source utilization patterns. Cultivation on different Biolog wells, along with next-generation sequencing, was then used to explore microbial responses to these substrates.

The present study was designed to investigate the metabolic profiles of the whole microbial community, including carbohydrates and amino acids, between caries-free (CF) children and children with S-ECC, including a thorough assessment of their responses to sucrose, lactose, and phenylalanine, thereby enhancing our understanding of the etiology of S-ECC.

## Materials and methods

### Ethics statement

The design and protocol of this study were approved by the Biomedical Ethics Committee of the Peking University School and Hospital of Stomatology (PKUSSIRB-201631129). Written informed consent was obtained from the parents or guardians of all participants before enrollment.

### Subjects

Children with S-ECC and CF children were recruited from five urban kindergartens in Beijing, China, and enrolled from patients at the Department of Pediatric Dentistry, Peking University School and Hospital of Stomatology. The term ‘Severe Early Childhood Caries (S-ECC)’ refers to the definition from the American Academy of Pediatric Dentistry (AAPD) [32]. Any subject who met the following criteria was excluded from the study: Having a systemic disease, having fewer than 18 teeth, having received antibiotics or topical fluoride treatment within 1 month, or having oral appliances. Caries status was charted using the dmft index, according to the codes proposed by the World Health Organization’s Oral Health Surveys Basic Methods (1997). Those with white spots on their teeth were excluded. Consistency in examination and caries diagnosis was ensured by the provision of training for dental examiners before the initiation of the study. The kappa value for intra-examiner agreement in the diagnosis of caries was 0.834.

### Sample collection

All participants were instructed to refrain from consuming food and drink for 2 h and to avoid rinsing their mouths before sample collection. The teeth were isolated using cotton rolls and gently air-dried. A supragingival plaque sample was collected and pooled from sound smooth surfaces of all teeth using a sterile dental excavator. Plaque samples were released from the excavator by agitation into a sterile, labeled 1.5-mL sterile centrifuge tube (Axygen, Union City, CA, USA) containing 1 mL phosphate-buffered saline (0.01 M, pH 7.2–7.4; Solarbio, Beijing, China). All specimens were collected within 5 min from 10:00 to 11:00 am, immediately put on ice, and then transported to the microbiology laboratory at Peking University School and Hospital of Stomatology within 2 h. The collected supragingival plaque samples were split into two aliquots. An aliquot of each sample was stored at – 20°C before further sequencing analysis. The other aliquot was used for Biolog analysis.

### Biolog assays and data analysis

The Biolog procedures and data analyses were performed according to the protocols described in previous studies [26,27]. The collected dental plaque samples were re-suspended in 12 mL of AN inoculating fluid (AN-IF, Catalog No. 72007; Biolog Inc., Hayward, CA, USA) and vortexed thoroughly for 1 min. Each plaque suspension was inoculated into a Biolog AN microplate (Catalog No. 1007; Biolog Inc.) at 100 µL per well. The Biolog AN microplate is a 96-well plate containing 95 sole carbon sources (wells A2-H12) and a blank well with water only (well A1). The plates were sealed with an anaerobic sachet bag and incubated in a 5% CO_2_ incubator at 37°C for up to 4 days. The optical density (OD) at 590 nm in each well was recorded at 24, 48, 72, and 96 h during incubation using a Biolog MicroStation (Biolog OmniLog version 4.1).

The overall metabolic activity of the oral microbiota was assessed using average well color development (AWCD), which was calculated using the following formula:

$$AWCD=\overset{n}{\underset{i=1}{\sum }}\frac{c_{i}}{n}$$

where C represents the corrected OD value in each well and n is the number of substrates (n = 95) for 95 sole carbon source wells. The corrected OD value was obtained by subtracting the OD value of the control well (A1) from the initial OD value of each carbon source well to correct for background activity. If the result was negative, the corrected OD value was deemed to be zero.

To avoid artificial differences (differences caused by varying initial OD values of the plaque suspensions, rather than by the pattern of carbon source consumption), the standardized OD values were used to compare the utilization of the sole carbon sources between two groups. The standardized OD values were obtained by dividing each corrected OD value by the AWCD of the plate, as proposed previously [26]. The Mann–Whitney U test (SPSS Statistics v 20, IBM Corp., Armonk, NY, USA) was used to compare the AWCD and sole carbon source usages between the CF and S-ECC groups.

### Microbial community profiles

To further explore the changes in the microbial community after metabolizing sucrose, lactose, and phenylalanine, plaque suspension in water (A1), sucrose (D11), α-D-Lactose (C4), and L-Phenylalanine (H2) after 96 h incubation were collected separately. Together with the original aliquot of the plaque before Biolog analysis (O), five separate samples of each subject (145 in total) were finally subjected to sequencing analysis.

Sequencing of the 16 S ribosomal RNA (rRNA) gene V3–V4 region was performed by Realbio Technology Co., Ltd (Shanghai, China). Briefly, the quality of the isolated microbial genomic DNA was evaluated using an ultraviolet spectrophotometer and electrophoresis through 1% agarose gels. PCR amplification of the V3–V4 region of the bacterial 16 S rRNA gene was performed using universal primers 341 F (5′- ACTCCTACGGGRSGCAGCAG −3′) and 806 R (5′- GGACTACVVGGGTATCTAATC −3′) incorporating sample barcode sequences. The PCR reactions were carried out under the following conditions: 95°C for 3 min; 30 cycles of 98°C for 20 s, 58°C for 15 s, and 72°C for 20 s; and 72°C for 5 min. The PCR products were separated using 2% agarose gel electrophoresis and purified using an Agencourt AMPure XP kit (Beckman Coulter, Inc., Brea, CA, USA). Sequencing was performed using the Illumina Hiseq PE250 sequencing platform (Illumina, Inc., San Diego, CA, USA) according to the manufacturer’s recommendations.

Raw data were demultiplexed and quality filtered using the QIIME pipeline (v1.9.1) [33]. To obtain high-quality sequences for the downstream analysis, sequences that were <220 bp or >500 bp, contained one or more ambiguous base calls (N), or had average quality scores <20 were removed. Chimeras were identified and removed using the UCHIME algorithm [34]. High-quality sequences were then clustered into operational taxonomic units (OTUs) at a 97% similarity cutoff, using the closed-reference OTU picking strategy (pick_closed_reference_otus.py) in QIIME. The expanded Human Oral Microbiome Database (eHOMD, v15.1) [35] was used to assign sequences to specific microbial taxonomies. Singleton OTUs were removed before further analyses.

Alpha diversity was estimated as microbial richness (Chao 1 index), evenness (Equitability index), and diversity (Shannon index). The relative abundances of microbial taxa at the phylum, class, order, family, genus, and species levels were calculated and compared. Differences in the α diversity indices and microbial relative abundances between the CF and S-ECC groups were calculated using the Mann–Whitney U test in IBM SPSS Statistics (v 20). The Wilcoxon test was used to examine the differences between O and A1 groups. The Friedman test was used to examine differences in α diversity indices and relative abundances among A1, D11, C4, and H2 groups. Post hoc analysis with the Wilcoxon signed-rank tests was conducted with a Bonferroni correction applied using R (v3.4.0). Principle coordinate analysis (PCoA) was performed based on the weighted UniFrac distance to visualize similarities among the microbial structures. The nonparametric method Adonis (n = 999) was further employed to examine the community differences among the sample groups. *P* values were corrected for multiple testing using the false discovery rate (FDR). Adonis components were used to build a dendrogram using the neighbor-joining algorithm implemented in Qiime and Geneious Prime 2019 [29].

### Data availability

The datasets analyzed for this study can be found in the NCBI Sequence Read Archive (SRA) database under Accession Number SRP222030. All other relevant data are within the manuscript and Supporting Information files.

## Results

### Study population

A total of 29 children aged 32–56 months were recruited in this study, including 15 children who were free of caries (dmft = 0, CF group) and 14 individuals with S-ECC (S-ECC group). The dmft and decayed, missing, and filled surfaces (dmfs) values were 14.36 ± 1.781 and 27.79 ± 9.649, respectively. The demographic and clinical characteristics of the subjects are displayed in Table 1 and detailed in Table S1. Student’s t tests and Chi-squared tests showed no significant differences between the S-ECC and CF groups with regard to age (*P* = 0.262) or sex (*P* = 0.573). A total of 29 supragingival plaque samples were obtained for Biolog analysis and 145 for sequencing analysis.

**Table 1.** Demographic and clinical characteristics of study population.

Items	CF group	S-ECC group
Sample size	15	14
Gender (male/female)	8/7	6/8
Age (months ±SD)	43.9 ± 6.0	41.3 ± 6.1
Decayed, missing, and filled primary teeth (dmft)	0	14.4 ± 1.7
Decayed, missing, and filled surfaces (dmfs)	0	27.8 ± 9.6

### Differences in carbon source usage by dental plaque between CF children and children with S-ECC

**Figure 1.**
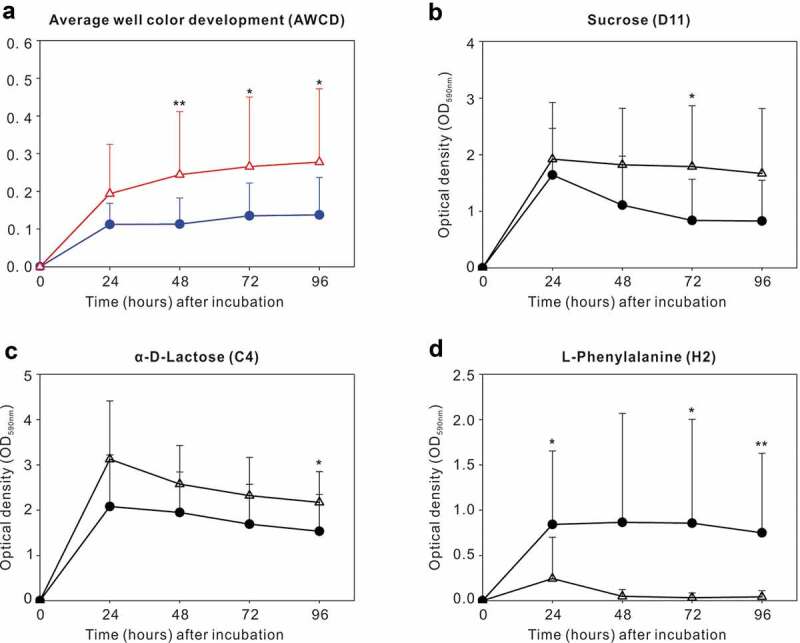
Carbon source utilization by dental plaque in the caries-free (●) and S-ECC (Δ) groups. (a) Average well color development (AWCD) with incubation time in the two groups. (b–d) Catabolic kinetics based on incubation time of sucrose (D11), α-D-Lactose (C4), and L-Phenylalanine (H2) in the caries-free and S-ECC groups. * *P* < 0.05 and ** *P* < 0.01 by the Mann–Whitney U test.

During the first 24 h after inoculation, no significant difference in the overall AWCD was found between the CF and S-ECC groups. At 48, 72, and 96 h, however, greater AWCD was noted in the S-ECC group, reflecting the fact that the S-ECC group showed greater metabolic responses compared with that of the CF group during later incubation periods (Figure 1(a)). The analyses of 95 sole carbon source usage patterns showed 12, 10, 11, and 16 significantly different carbon sources between the two groups at 24, 48, 72, and 96 h, respectively. Additional data are provided in Supplementary Table S2.

We then explored sole carbon source utilization patterns of sucrose (D11), α-D-Lactose (C4), and L-Phenylalanine (H2), between the two groups at 24, 48, 72, 96 h, respectively. S-ECC group had greater standardized OD values than the CF group in sucrose at 72 h and in α-D-Lactose at 96 h. Except for these observations, no significant differences were identified between the two groups at other time points when using sucrose or α-D-Lactose as the sole carbon sources (Figure 1(b,c)). For L-Phenylalanine, however, the CF group had more active metabolic responses than the S-ECC group at 24, 72, and 96 h (Figure 1(d)).

### Overall sequencing data

A total of 145 supragingival dental plaque samples (five samples per subject) were collected and underwent further high-throughput sequencing analysis. After quality control, an average of 24 157 sequences per sample (ranging from 19 409 to 29 698) were generated. These high-quality sequences were finally clustered into 548 non-singleton OTUs, with 80–278 OTUs in individual specimens (Table S1). Assigning reference sequence OTUs to taxonomy using eHOMD, a total of 10 phyla, 22 classes, 37 orders, 62 families, 129 genera, and 446 species were detected from all samples.

### Microbial profiles in the CF and S-ECC groups

We first compared and characterized the microbiome in the original plaque (O) between the CF and S-ECC groups to gain insight into microbial diversity and structure in the oral cavity of these 3–5 year old children with or without S-ECC, which was also used as the baseline for further analysis.

**Figure 2.**
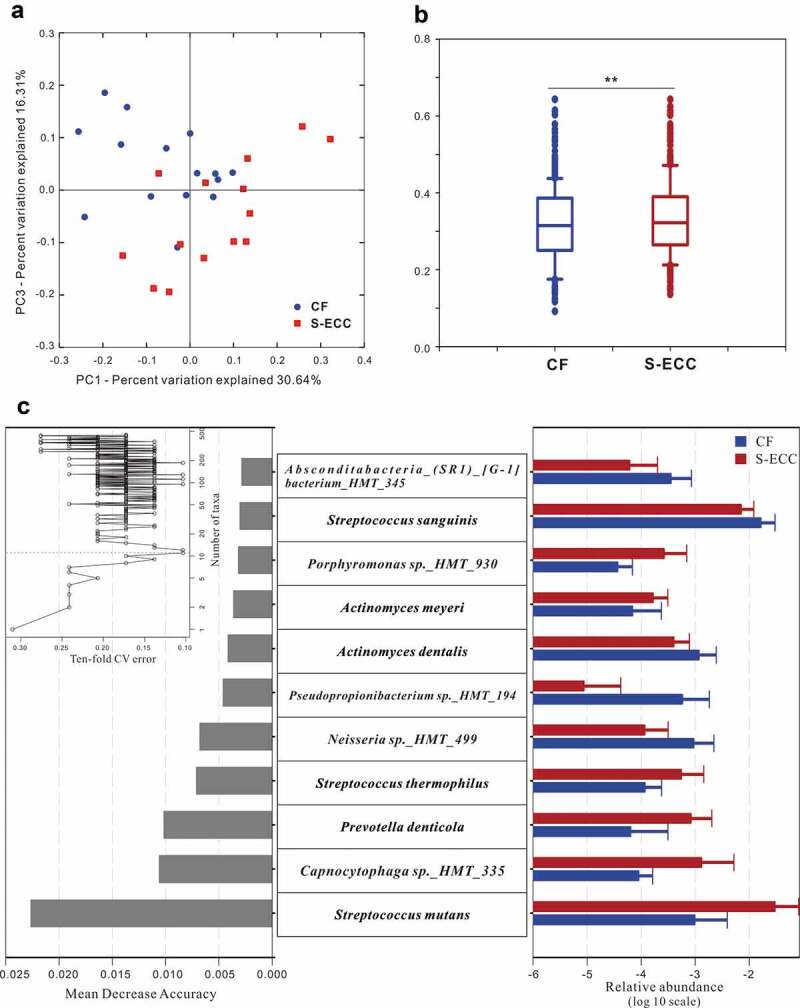
Microbial communities of caries-free (CF) and S-ECC in original plaques before being cultured. (a) A principal coordinate analysis (PCoA) based on the weighted UniFrac distances. (b) The weighted UniFrac distance values of the CF and S-ECC groups. All data are presented as medians and the 10th, 25th, 75th, and 90th percentiles. **P < 0.01 as assess by the nonparametric method Adonis (n = 999). (c) Microbial indicators for classification of S-ECC status. The 11 most discriminatory species were identified by applying Random Forest models of their relative abundances in the original plaque between the CF and S-ECC groups. Species are shown in ascending order of their importance to the accuracy of the model. Importance was determined based on the Mean Decrease Accuracy (left) produced by the ‘randomForest’ package in R (v3.4.0). The inset of the left part shows the misclassification/error rate as a function of the number of input species. The right part shows the log10-transformed relative abundances of the 11 most discriminatory species in the CF (blue) and S-ECC (red) groups.

Alpha diversity (diversity within a given community), including richness (the total number of taxa), evenness (distribution of relative species abundances), and diversity (quantitative species-based measures which is positively correlated with species richness and evenness) [36], of the microbial community showed no significant differences between the CF and S-ECC groups. However, PCoA of beta diversity (partitioning of diversity among communities) based on the weighted UniFrac distances exhibited segregations between the two groups. The Adonis test confirmed this dissimilarity in the microbial community structures between the CF and S-ECC groups (Figure 2(a,b)).

Random Forests models were then trained to construct microbial indicators of S-ECC. Using the profiles of species, the performance of models based on the microbiota was further evaluated using a 10-fold cross-validation approach. According to the ‘rfcv’ function in the randomForest package, the 11 top-ranking important taxa had the most discriminatory power and led to reasonably good classification of S-ECC status (misclassification/error rate: 10.34%). These 11 species included *Streptococcus mutans* and *Prevotella denticola* which were more abundant in S-ECC group, and *Neisseria sp._HMT_499* and *Streptococcus sanguinis* which were more abundant in CF group (Figure 2(c)).

### Influence of anaerobic culture on the microbiota using Biolog AN microplates

During the Biolog analysis, plaque samples were cultured anaerobically for 96 h on 96-well AN microplates. Sequences of original plaque (O) and plaque in well A1, a blank well with water only, were compared to explore the effect of anaerobic culture on the microbiota of the CF and S-ECC samples.

After being cultured anaerobically in water, significant differences in the microbial structure were observed in both the CF and S-ECC samples (Adonis test for weighted UniFrac distances, *P* < 0.01 in CF and *P* < 0.05 in S-ECC, Figure S1). In the CF group, the richness index showed no significant difference between O and A1. The equitability and Shannon indices demonstrated that A1 was significantly less even and diverse than the O microbial community. For the S-ECC samples, however, the richness, evenness, and diversity of the microbial community demonstrated no significant difference between O and A1 (Table 2).

**Table 2.** Alpha diversity comparison between original plaque (O) and well A1 (P values of the Wilcoxon test).

Groups	Shannon (diversity index)	Chao 1 (richness index)	Equitability (evenness index)
CF	*P* = 0.005	*P* = 0.496	*P* = 0.005
S-ECC	*P* = 0.594	*P* = 0.551	*P* = 0.551

CF, caries-free; S-ECC, severe early childhood caries.

Among the detected species, 131 taxa (29.37%) of CF group and 117 taxa (26.23%) of S-ECC group showed differences in their relative abundance between O and A1, respectively, of which 82 species were common between CF and S-ECC groups. Compared with the original plaque (O), 72 species from the CF group, such as aerobic *Neisseria subflava*, were less abundant in A1, whereas 59 taxa were enriched in A1, including some obligately or facultatively anaerobic bacteria like *Prevotella maculosa* and *Abiotrophia defectiva*. In the S-ECC group, 68 taxa were less abundant and 49 were more abundant after being cultured in A1, suggesting that anaerobic culture had no significant influence on the number of species and their abundances were similar; however, complex changes in the taxa within the S-ECC community still existed (Table S3).

### Plaque microbiota had related but distinct responses when metabolizing lactose and sucrose as sole carbon sources

**Figure 3.**
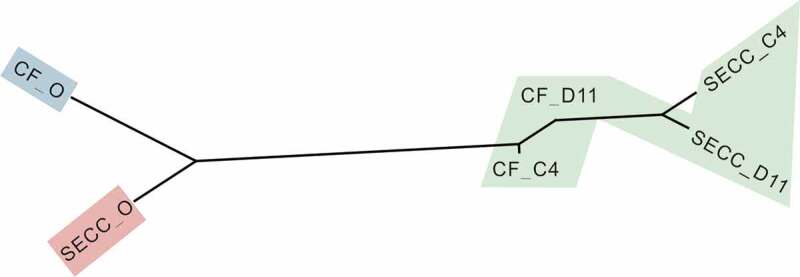
Microbial communities of CF and S-ECC plaques before and after sucrose and lactose consumption. Neighbor-joining phylogeny based on pairwise Adonis components for beta diversity. Branch lengths represent the degrees to which the bacterial communities are differentiated. Groups shaded in green showed no significant differences in beta diversity values. CF, caries-free children; S-ECC, severe early childhood caries. O, original aliquot of plaque before Biolog analysis; D11, sucrose; C4, α-D-Lactose.

A neighbor-joining dendrogram was constructed using the variance components obtained from the pairwise Adonis test (Figure 3 and Table 3). The pairs of CF_D11-S-ECC_D11, CF_D11-CF_C4, and S-ECC_C4-S-ECC_D11 groups were connected by short branch lengths and were not significantly separated. All remaining comparisons among the different groups (plaque communities) were significant. Overall, in response to sucrose, the originally different microbial communities of the S-ECC and CF plaques both changed markedly, eventually becoming similar to each other (*P* = 0.1546). In response to lactose, however, the microbial communities of the S-ECC and CF plaques remained dissimilar (*P* = 0.0109).

**Table 3.** Pairwise Adonis analysis for significant differences in beta diversity measures (weighted UniFrac distance) among the different groups of original plaque (O), D11, and C4 plaque samples^a.^

	*P* values or *F* values					
Group	CF_O	S-ECC_O	CF_D11	S-ECC_D11	CF_C4	S-ECC_C4
CF_O		0.0067**	0.0019**	0.0019**	0.0019**	0.0019**
S-ECC_O	3.708		0.0019**	0.0019**	0.0019**	0.0019**
CF_D11	7.6726	7.4603		0.1546	0.3840	0.0113*
S-ECC_D11	13.088	10.769	1.6557		0.0105*	0.3439
CF_C4	7.6734	7.6045	1.0236	3.2687		0.0109*
S-ECC_C4	11.916	9.8966	3.2754	1.2037	3.2335	

^a^The top right values are *P* values corrected using the false discovery rate (FDR). The bottom left values are *F* values (variance components). * *P* < 0.05, ** *P* < 0.01. CF, caries-free; S-ECC, severe early childhood caries children. O, original aliquot of plaque before biological analysis; D11, sucrose; C4, α-D-Lactose.

**Figure 4.**
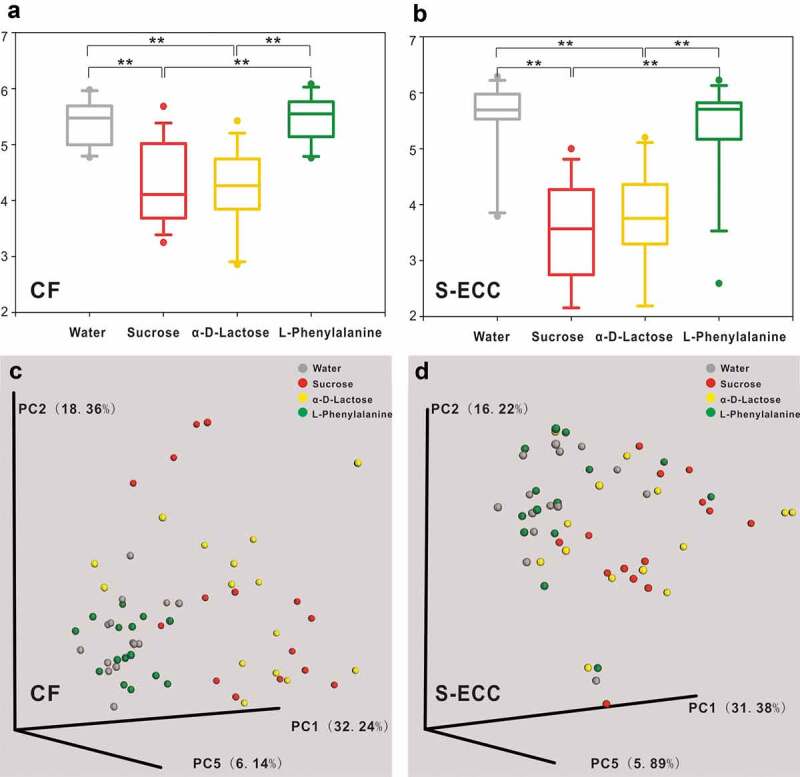
Alpha and beta diversity of microbial communities when using water, sucrose, α-D-Lactose, and L-Phenylalanine as the sole carbon source. Boxplots of Shannon indices (alpha diversity estimator) in the CF (a) and S-ECC (b) groups. All data are presented as medians and the 10th, 25th, 75th, and 90th percentiles. ** *P* < 0.01 as assessed using the post hoc analysis for the Friedman test in SPSS (v 20). A principal coordinate analysis (PCoA) of beta diversity based on the weighted UniFrac distance values in the CF (c) and S-ECC (d) groups.

In both the CF and S-ECC groups, compared with the microbiota cultured in water (A1), the microbial community richness, evenness, and diversity all significantly decreased in response to lactose (C4) and sucrose (D11) as the sole carbon sources; however, there was no significant difference between lactose and sucrose (Figure 4(a,b), Table S4). Weighted UniFrac distance-based analysis showed that the microbial communities of the C4 and D11 samples were dissimilar from those of A1, but were not well differentiated from each other (Figure 4(c,d), Table S4).

**Figure 5.**
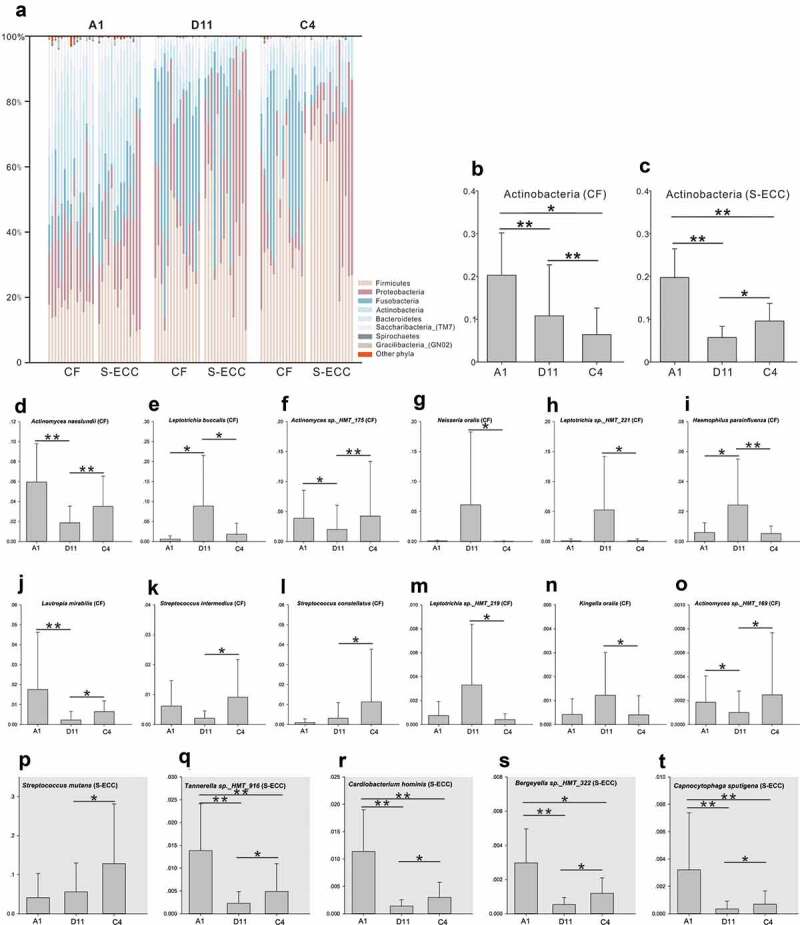
Microbial communities of lactose and sucrose. (a). Relative abundances of the resident bacterial phyla under different carbon sources. (b, c). Differently abundant phylum Actinobacteria between sucrose and α-D-Lactose in the CF and S-ECC groups. (d–o) Differently abundant species between sucrose and α-D-Lactose in the CF group. (p–t) Differently abundant species between sucrose and α-D-Lactose in the S-ECC group. Species are listed in descending order according to the mean relative abundance of A1, D11, and C4. Bars indicate mean relative abundances with standard deviations. The significance of the differences among the groups was determined using the Friedman test. Post hoc analysis with Wilcoxon signed-rank tests was conducted with a Bonferroni correction applied. *P < 0.05, **P < 0.01.

When using sucrose or lactose as the carbon source, the microbial community structure was quite different from that in water at the phylum level (Figure 5(a)). However, between sucrose and lactose, plaque samples had similar microbial compositions, except for the Actinobacteria, which were more abundant in lactose than that in sucrose, in both the CF and S-ECC groups (Figure 5(b,c)).

At the species level, 71 species in the CF group and 109 species in the S-ECC group showed significant differences in their relative abundance in sucrose compared with that in water (Table S5), whereas there were 65 and 95 differentially abundant species in CF and S-ECC, respectively, between lactose and water (Table S6). Most of the differently distributed species (CF: 62/71 species and S-ECC: 104/109) demonstrated decreased abundance in lactose compared with those in the blank samples, which was similar to the results for the taxa in sucrose (CF: 58/65 species and S-ECC: 91/95). When further exploring the microbiota between sucrose and lactose, out of 12 differentially abundant species in CF, two *Streptococcus* species, three *Actinomyces* species, and *Lautropia mirabilis* were more abundant, and *Neisseria oralis*, three *Leptotrichia* species, *Haemophilus parainfluenzae*, and *Kingella oralis* were less abundant in lactose (Figure 5(d–o)). In the S-ECC group, however, five differentially abundant species, including *Streptococcus mutans, Tannerella sp._HMT_916, Cardiobacterium hominis, Bergeyella sp._*HMT*_322*, and *Capnocytophaga sputigena*, all showed higher relative abundance in lactose than in sucrose (Figure 5(p–t)).

### CF and S-ECC microbiota when using phenylalanine as the sole carbon source

The beta diversity analysis showed there was no clear separation between the microbial communities of phenylalanine and water. The richness, evenness, and diversity of the microbiota under phenylalanine were also not significantly different from those of the blank control, but were greater than those of sucrose and lactose, in both the CF and S-ECC groups (Table S4).

The great variations in community structure between sucrose and lactose meant that the taxa analyses could be focused on the comparisons between the microbiota under phenylalanine and water. In the CF group, the phylum Gracilibacteria GN02 was more abundant in phenylalanine than in water. Other phyla of the CF group and all phyla of the S-ECC group were not significantly different between phenylalanine and water. At the species level, the differentially abundant species of the CF group included *Gemella morbillorum, Pseudopropionibacterium propionicum, Saccharibacteria_(TM7)_[G-6] bacterium_HMT_870, Neisseria sp._*HMT*_018*, and *Alloprevotella sp._*HMT*_912*, which were enriched in response to phenylalanine. In the S-ECC group, only *Haemophilus sp._HMT_036* was differentially abundant species, with a higher relative abundance in water than in phenylalanine.

## Discussion

S-ECC and healthy plaques harbored a distinct microbial community with varying metabolic capacities. Previous studies have indicated that plaques with active caries displayed a lower plaque pH and a longer recovery time after exposure to sugars [37,38]. But, before and after change of pH, how the entire community of S-ECC interacts with carbohydrates is still not fully understood since existing conclusions often come from studies on individual oral species. Therefore, we aimed to fill in these knowledge gaps by analyzing genomic and metabolic profiles of S-ECC plaques in their interaction with carbohydrates at a community-level. S-ECC plaques displayed enhanced capability to utilize carbohydrates, like sucrose and lactose, comparing to CF ones (Figure 1), possibly due to overabundances of acidogenic species in S-ECC microbial community before being cultured, like *S. mutans, Capnocytophaga* species and *P. denticola* (Figure 2) which have ability to metabolize carbohydrates [10,39,40]. In subsequent phase, Figure 3 shows that distances of community structure between carbohydrates and original plaque in S-ECC group were longer than those of CF group. These findings, together with the observation that S-ECC plaques had more differently distributed species after metabolizing carbohydrates than CF plaques, underscored that S-ECC plaques had greater response to carbohydrates. One of the reasons may be that CF plaques contained significantly higher levels of alkali-generating species, such as *S. sanguinis* (Figure 2) which is capable of producing alkali via the arginine deiminase system and neutralizing pH [41,42], protecting the whole community from acid stress and finally enabling CF plaques be less influenced by carbohydrates than S-ECC plaques. Taken together, these results showed that in contrast with healthy ones, S-ECC plaques could utilize carbohydrates more actively and then had greater response to them, and consequently, the effect of carbohydrates on S-ECC plaques would be amplified. Strategies that return ‘disease’ microbiota to ‘healthy’ one are emphasized and, therefore, could maintain the beneficial properties of normal microflora and inhibit the development of dental caries.

Biolog technology was used to characterize community-level metabolic profile of caries in children. Previous studies reported that ECC patients displayed greater overall metabolic activity than that of healthy group [27], which was consistent with results in the present study. Not only that but we further used biolog microplates as growth media with separate carbon source. Effects of each carbon source on microbiota can thus be determined under defined but controllable conditions [43].

We detailed ecological microbial shift subsequent to sucrose challenge to serve as a positive control of microbial responses to carbon sources. Previous studies mainly focused on the effects of sucrose on one single species or artificial biofilm [11,44,45]. Instead, we sampled supragingival plaque directly from oral cavity, trying to get closer to microbial reactions to sucrose in an *in-vivo* situation. Our results showed that the majority of differently distributed taxa were suppressed after sucrose consumption while the abundances of *S. gordonii, S. parasanguinis*, and *S. sanguinis* increased remarkably, in consistent with the previous study which also reported elevated proportions of non-mutans Streptococci after frequent consumption of sucrose [46]. According to the ecological plaque hypothesis [47,48], this is probably because conditions of low pH resulting from sucrose metabolism affect the balance of the resident microflora and favor the proliferation of bacteria which could tolerant acid environment. Even less acid-tolerant than *S. mutans*, non-mutans Streptococci also could utilize sucrose to survive when facing with low pH conditions, as the phenotypic adaptation occur and thus enhance their acid tolerance [49].

Lactose is a disaccharide formed by galactose and glucose, and can be fermented yielding organic acids and ultimately cause a decaying lesion [50]. Similar to the compositional changes in response to sucrose, a remarkable increase of non-mutans Streptococci which efficiently utilize lactose and contributed to acidification [51], was also observed in lactose consumption. More notably, distinct patterns of microbial response to lactose and sucrose were identified at the levels of population abundance and community structure. For example, our results showed that lactose led to less differently distributed taxa than sucrose did in both CF and S-ECC groups. This may be due to a smaller drop in plaque pH caused by lactose than sucrose [52]. Some species, such as less acid tolerant bacteria, could survive in lactose metabolism but would be suppressed by such a dramatic pH fall generated by sucrose metabolism. Additionally, sucrose made originally different S-ECC and CF communities eventually become similar to each other (Figure 3), indicating that buffer capacity of healthy plaque was insufficient to resist acid stress caused by sucrose metabolism and eventually shift towards pathogenic state. In the metabolism of lactose, however, dissimilarity of microbial structure between CF and S-ECC plaques was maintained, suggesting that lactose has relatively smaller impact on microbial community than sucrose. Previous studies indicated that lactose was less cariogenic than sucrose, based on animal studies and intraoral test [50]. Our results provided evidence and supported this view from the aspect of the microbial community structure.

Our study demonstrated that CF group metabolized phenylalanine more actively than S-ECC group, suggesting that phenylalanine might be related to caries free state. Previous studies also suggested that phenylalanine could have an inhibitory effect on caries development [23,24]. In bacteria, phenylalanine may first be deaminated then convert to phenylpropionate or phenylacetate, both generating alkali [53] which is an essential factor in maintaining plaque pH homeostasis. So we additionally analyzed the plaque microbiota cultured in phenylalanine well to reveal the microbial community structure and explore caries suppression potential of phenylalanine. The differentially abundant species of CF and S-ECC groups in response to phenylalanine have no reported connections to phenylalanine metabolism, probably because studies of the effect of phenylalanine on the oral microbiota are very limited. A previous study showed that phenylalanine was essential for the growth of oral anaerobes, such as *Capnocytophaga gingivalis, Eubacterium timidurn, Fusobacterium nucleatum, Porphyromonas gingivalis, Treponema denticola*, and *Treponema vincentii* [25]. However, none of these species showed a difference in relative abundance in response to phenylalanine compared with that to water in our results. Therefore, the relationship between phenylalanine and the S-ECC microbiota requires further study.

Here we reported that S-ECC plaques displayed higher overall metabolic activity compared to CF ones, including enhanced capability to utilize cariogenic carbohydrates like sucrose and lactose, and had greater response to sugars in prolonged acidification. Our work suggested that approaches that return microbiota from disease state to healthy one were of crucial importance in S-ECC control. And in this way, S-ECC children could benefit from the normal microflora and inhibit the development of dental caries. Our data verified although both are cariogenic, lactose has less cariogenicity than sucrose from microbial community structural aspect. We also proposed caries suppression potential of phenylalanine, but future surveys with the mechanisms, such as metabolic pathways of phenylalanine in oral bacteria and its role in biofilm formation, are needed. This study will improve our understanding of the interaction between microbial communities and environmental factors in S-ECC.

## Supplementary Material

Supplemental Material
