# Supplementary figures and images for: Carbon source utilization patterns in dental plaque and microbial responses to sucrose, lactose, and phenylalanine consumption in severe early childhood caries
Source: J Oral Microbiol. 2020 Jun 23;12(1):1782696. doi: 10.1080/20002297.2020.1782696 (PMC7482870; doi:10.1080/20002297.2020.1782696)

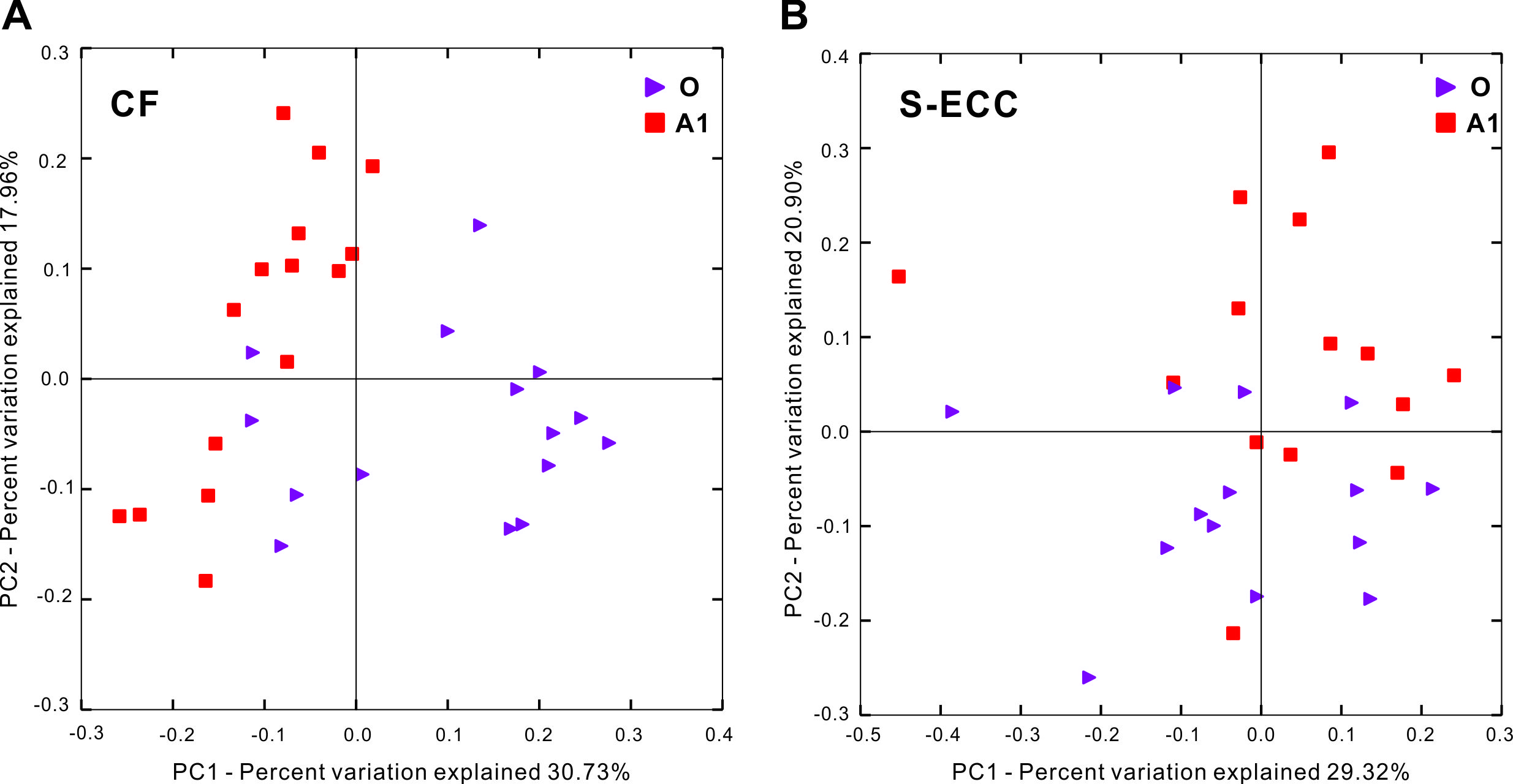

Supplement: Supplemental Material [file ZJOM_A_1782696_SM9340.zip › supplementary/Figure S1.jpg]
