# Supplementary material for: Carbon source utilization patterns in dental plaque and microbial responses to sucrose, lactose, and phenylalanine consumption in severe early childhood caries
Source: J Oral Microbiol. 2020 Jun 23;12(1):1782696. doi: 10.1080/20002297.2020.1782696 (PMC7482870; doi:10.1080/20002297.2020.1782696)
Supplement: Supplemental Material [file ZJOM_A_1782696_SM9340.zip › supplementary/Table S1.doc]

**Table S1.**  Demographic characteristics of subjects and general information of sequencing results

| **Sample**  **ID** | **Gender** | **Age (month)** | **dmft** | **dmfs** | **Final sequences** | | | | | **OTU^a^ num** | | | | |
| --- | --- | --- | --- | --- | --- | --- | --- | --- | --- | --- | --- | --- | --- | --- |
|  |  |  |  |  | **Original sample**  **(O)** | **Water (A1)** | **Sucrose (D11)** | **α-D-Lactose (C4)** | **L-Phenylalanine (H2)** | **Original sample**  **(O)** | **Water (A1)** | **Sucrose (D11)** | **α-D-Lactose (C4)** | **L-Phenylalanine (H2)** |
| CF01 | Female | 52 | 0 | 0 | 22247 | 25581 | 24889 | 24691 | 23800 | 234 | 239 | 168 | 197 | 219 |
| CF02 | Male | 46 | 0 | 0 | 22339 | 24250 | 22637 | 21867 | 24574 | 192 | 189 | 161 | 177 | 182 |
| CF03 | Male | 38 | 0 | 0 | 27274 | 23219 | 25663 | 27782 | 21628 | 223 | 207 | 181 | 191 | 207 |
| CF04 | Female | 34 | 0 | 0 | 25075 | 20475 | 25825 | 27107 | 24880 | 231 | 206 | 162 | 173 | 227 |
| CF05 | Female | 35 | 0 | 0 | 26562 | 25157 | 29406 | 29649 | 24084 | 212 | 193 | 168 | 167 | 202 |
| CF06 | Male | 53 | 0 | 0 | 25770 | 26144 | 24876 | 23815 | 19852 | 203 | 188 | 125 | 165 | 188 |
| CF07 | Male | 51 | 0 | 0 | 25614 | 25430 | 26524 | 23354 | 27681 | 192 | 210 | 148 | 121 | 228 |
| CF08 | Male | 45 | 0 | 0 | 23223 | 25817 | 23865 | 23646 | 24573 | 180 | 177 | 170 | 149 | 177 |
| CF09 | Female | 43 | 0 | 0 | 23679 | 23869 | 27136 | 22238 | 24139 | 262 | 231 | 230 | 198 | 244 |
| CF10 | Male | 39 | 0 | 0 | 24253 | 24919 | 22870 | 27592 | 25216 | 274 | 269 | 236 | 238 | 273 |
| CF11 | Female | 40 | 0 | 0 | 22320 | 24814 | 27954 | 29698 | 26316 | 243 | 240 | 217 | 202 | 246 |
| CF12 | Male | 41 | 0 | 0 | 23124 | 24093 | 23494 | 24208 | 21907 | 219 | 220 | 177 | 161 | 203 |
| CF13 | Female | 49 | 0 | 0 | 24688 | 25462 | 22174 | 25626 | 22918 | 254 | 211 | 216 | 212 | 204 |
| CF14 | Female | 47 | 0 | 0 | 24855 | 27204 | 26636 | 25486 | 25928 | 254 | 245 | 233 | 230 | 223 |
| CF15 | Male | 45 | 0 | 0 | 24710 | 20641 | 24202 | 20524 | 23076 | 247 | 246 | 211 | 214 | 247 |
| S-ECC01 | Female | 44 | 16 | 46 | 23531 | 25314 | 22461 | 20087 | 23184 | 259 | 278 | 213 | 231 | 259 |
| S-ECC02 | Male | 43 | 14 | 31 | 24505 | 22290 | 22674 | 22909 | 22017 | 234 | 228 | 195 | 181 | 243 |
| S-ECC03 | Male | 32 | 17 | 35 | 21870 | 24699 | 21229 | 21180 | 25800 | 221 | 233 | 177 | 180 | 233 |
| S-ECC05 | Female | 38 | 15 | 18 | 22521 | 26104 | 27840 | 25526 | 24813 | 238 | 267 | 159 | 196 | 267 |
| S-ECC06 | Male | 37 | 14 | 25 | 19409 | 22011 | 20669 | 20775 | 19940 | 159 | 161 | 124 | 126 | 166 |
| S-ECC07 | Female | 56 | 17 | 44 | 23890 | 26038 | 26516 | 24630 | 23363 | 237 | 237 | 166 | 185 | 235 |
| S-ECC08 | Male | 41 | 13 | 28 | 22441 | 23957 | 24417 | 23158 | 21578 | 200 | 200 | 156 | 148 | 208 |
| S-ECC09 | Female | 49 | 13 | 19 | 24787 | 24795 | 20635 | 23633 | 25582 | 261 | 254 | 176 | 196 | 239 |
| S-ECC10 | Female | 34 | 13 | 16 | 22013 | 23903 | 27359 | 22085 | 23349 | 238 | 229 | 169 | 172 | 217 |
| S-ECC11 | Male | 43 | 12 | 20 | 25592 | 25827 | 25840 | 22775 | 26451 | 240 | 243 | 161 | 80 | 232 |
| S-ECC12 | Female | 41 | 12 | 19 | 23536 | 26603 | 22609 | 22662 | 23786 | 242 | 220 | 117 | 183 | 228 |
| S-ECC13 | Male | 39 | 17 | 37 | 25046 | 21625 | 26305 | 23934 | 22893 | 268 | 259 | 228 | 229 | 246 |
| S-ECC14 | Female | 37 | 14 | 25 | 23096 | 25711 | 23349 | 24106 | 24689 | 211 | 197 | 138 | 136 | 201 |
| S-ECC15 | Female | 44 | 14 | 26 | 21422 | 23319 | 22392 | 23363 | 25603 | 219 | 199 | 123 | 84 | 186 |

^a^ The operational taxonomic units (OTUs) were defined with 3 % dissimilarity level.
